# Integrating information from historical data into mechanistic models for influenza forecasting

**DOI:** 10.1371/journal.pcbi.1012523

**Published:** 2024-10-30

**Authors:** Alessio Andronico, Juliette Paireau, Simon Cauchemez

**Affiliations:** 1 Mathematical Modelling of Infectious Diseases Unit, Institut Pasteur, Université Paris Cité, UMR2000 CNRS, Paris, France; 2 Infectious Diseases Department, Santé publique France, Saint-Maurice, France; The University of Hong Kong, CHINA

## Abstract

Seasonal influenza causes significant annual morbidity and mortality worldwide. In France, it is estimated that, on average, 2 million individuals consult their GP for influenza-like-illness (ILI) every year. Traditionally, mathematical models used for epidemic forecasting can either include parameters capturing the infection process (mechanistic or compartmental models) or rely on time series analysis approaches that do not make mechanistic assumptions (statistical or phenomenological models). While the latter make extensive use of past epidemic data, mechanistic models are usually independently initialized in each season. As a result, forecasts from such models can contain trajectories that are vastly different from past epidemics. We developed a mechanistic model that takes into account epidemic data from training seasons when producing forecasts. The parameters of the model are estimated via a first particle filter running on the observed data. A second particle filter is then used to produce forecasts compatible with epidemic trajectories from the training set. The model was calibrated and tested on 35 years’ worth of surveillance data from the French Sentinelles Network, representing the weekly number of patients consulting for ILI over the period 1985–2019. Our results show that the new method improves upon standard mechanistic approaches. In particular, when retrospectively tested on the available data, our model provides increased accuracy for short-term forecasts (from one to four weeks into the future) and peak timing and intensity. Our new approach for epidemic forecasting allows the integration of key strengths of the statistical approach into the mechanistic modelling framework and represents an attempt to provide accurate forecasts by making full use of the rich surveillance dataset collected in France since 1985.

## Author summary

Seasonal influenza causes significant morbidity and mortality worldwide. In France, on average, 2 million individuals consult their GP for influenza-like-illness (ILI) every year. Forecasting the future trajectory of an epidemic in real-time can inform public health responses. Traditionally, two types of mathematical models are used to forecast infectious diseases outbreaks: (1) mechanistic models that explicitly capture transmission mechanisms and (2) statistical models that rely on the similarity between epidemic dynamics over the years. In contrast to statistical models, mechanistic models usually do not use information on past seasons to inform forecasts. This may result in poor performance when forecasted trajectories are very different from past epidemics. Here, we propose a framework that combines these two approaches by allowing mechanistic models to learn from trends observed in past epidemics. We evaluate this approach in the context of seasonal influenza in France. Our results show that the new method improves upon standard mechanistic approaches. In particular, our model provides increased accuracy for predicting the epidemic trajectory one to four weeks into the future, as well as the timing and size of the peak. Our new approach for epidemic forecasting allows combining statistical and mechanistic models to improve forecasts performance.

## Introduction

Seasonal influenza causes significant annual morbidity and mortality worldwide and induces important stress on healthcare structures. In France, it is estimated that, every year, 2 million individuals on average consult their GP for influenza-like-illness (ILI), with mortality attributable to seasonal influenza estimated at 9000 deaths per year on average [1]. Epidemics occur during winter in France (November-March) as typically observed in countries with temperate climates. Every year, the epidemic dynamics can vary substantially in terms of intensity and timing. Forecasts of influenza outbreaks in real-time can inform public health response and help healthcare authorities to plan communication and vaccination campaigns, better anticipate overcrowding in healthcare structures and increase operational capacity to meet upsurges in demand.

Traditionally, mathematical models used to forecast infectious diseases outbreaks fall into two broad categories [2,3]. First, mechanistic models fitted to an ongoing epidemic aim to forecast its trajectory building on a mechanistic understanding of the transmission dynamics and its determinants (e.g. capturing the depletion of susceptibles, the dependence of transmission rates to climate variables…), either at the population level (compartmental models) [4–7] or at the individual level (agent-based models) [8,9]. Major developments have been made over the last decade to improve this approach. In particular, data assimilation, or filtering, methods used in conjunction with variations of the susceptible–infectious–recovered (SIR) model, showed good performance [10]. In contrast, phenomenological, also called statistical models, do not aim to explicitly capture transmission mechanisms. Instead, they rely on the realization that there is a certain degree of similarity between epidemic dynamics over the years. By training a statistical model to historical data, they can assess these repetitive patterns and build on that to propose forecasts. They include (but are not limited to) time-series models, such as autoregressive integrated moving average (ARIMA) models [11,12], that leverage the correlation structure of the data, and various types of regression models, such as generalized linear regression [13], generalized additive models (GAM) [14,15] or Gaussian process regression [16], that usually incorporate external predictors. Recent improvements in the statistical field include models that use different types of kernel conditional density estimation [3,17], a nonparametric statistical methodology that is a distribution-based variation on nearest-neighbors regression [18]. Machine-learning approaches are also increasingly used for infectious diseases forecasting, exploiting information from various external data sources [15,19–22]. Several multi-model comparisons of seasonal ILI forecasting in the United States have shown that, in practice, statistical models performed slightly better than mechanistic models for short-term forecasts [23–26]. In addition, recent years have seen the development of ensemble models applied to infectious diseases forecasting [15,26–30]. These models average forecasts over a range of individual models that can be mechanistic and statistical, and usually provide better forecast accuracy on average.

To improve the performance of ensemble models, we need to improve that of individual models they rely on. In contrast to statistical models that make extensive use of past epidemics during the calibration process, mechanistic models are usually independently initialized for each season and thus, do not use information on past seasons to inform forecasts. This may result in poor performance when forecasts contain trajectories that are vastly different from past epidemics. Here, we propose a framework to integrate information from other epidemic seasons into forecasts generated with mechanistic models. Our approach belongs to the family of filtering methods mentioned above, used in conjunction with a transmission model (typically an SIR model). The reader can refer to the paper by Yang et al [10] for an extensive description of these methods. Briefly, filtering methods can be used to estimate the state variables (e.g., number of susceptible persons) and infer the model parameters. They use the observations to recursively inform the model so that the trajectory of the observed epidemic curve is better matched by the model. The SIR model with inferred parameters and updated state variables, can then be propagated into the future to produce forecasts. Yang et al compared different filtering methods, three of which are included in this paper: a standard bootstrap particle filter (PF) and two ensemble filters [10]. As our approach builds on a PF, we present the key characteristics of the standard PF in the *Materials and Methods* section, and then describe how our approach extends the PF to integrate information from a subset of training seasons into forecasts. In short, when generating projections with a standard PF, the transmission model is used to update the particle trajectory but no weighting or resampling is done—since no observations are available for the future. We develop a new approach, referred to as a “modified particle filter” (mPF), to continue weighting and resampling particles while generating projections: the weights assigned to the particles are computed using information extracted from training seasons. The main idea is to give larger weights to trajectories that are closer to what was observed in training seasons, so that forecasted trajectories are compatible with trajectories observed in training seasons. We evaluate this approach in the context of seasonal influenza in France and show that it substantially improves performance compared to existing filters. We also investigate the minimum number of training seasons that are necessary for the approach to become relevant.

## Materials and methods

### Data

**Fig 1:**
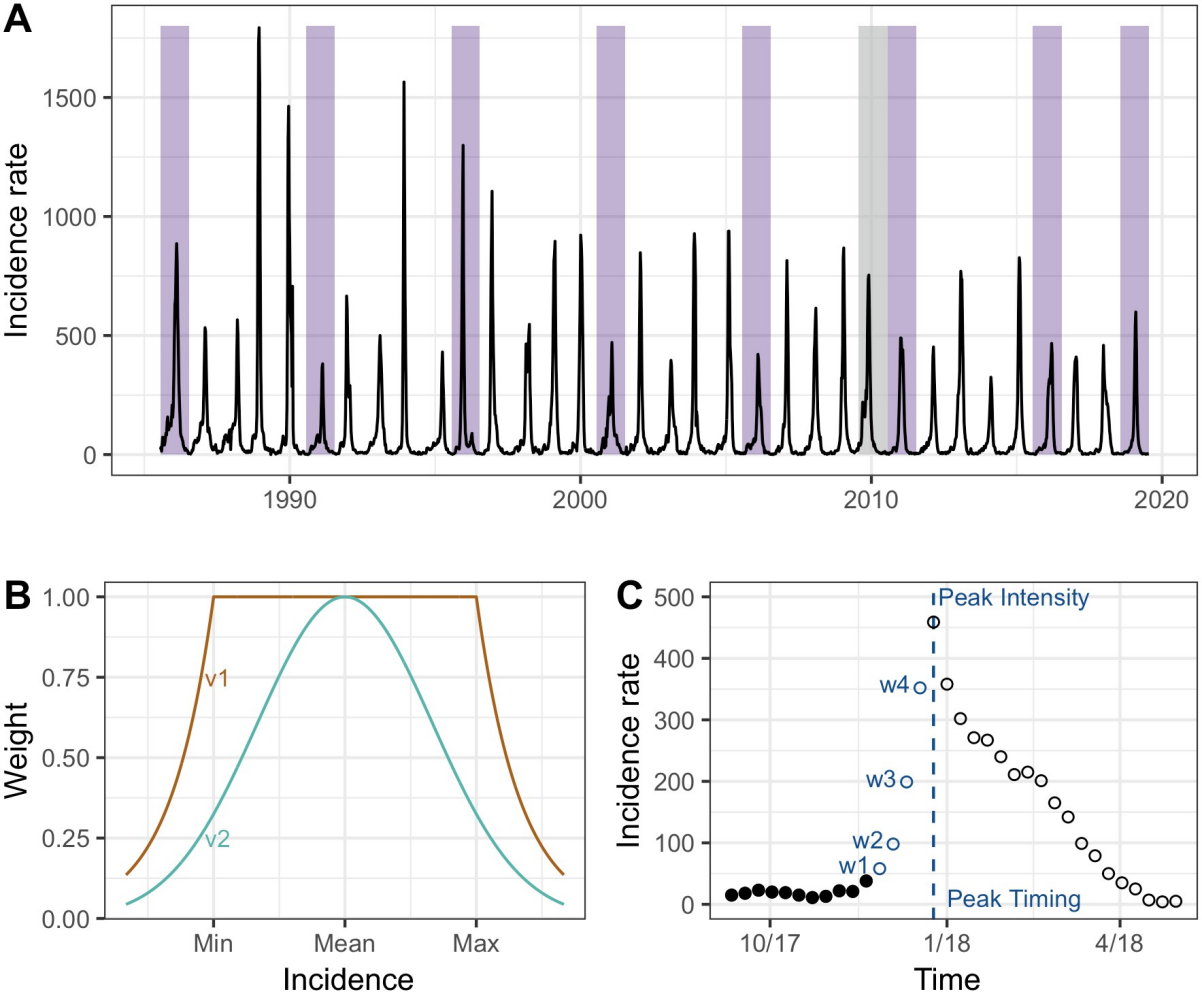
ILI time-series, weighting schemes and projection targets. **A**. Time series used for the analyses and representing ILI incidence rate (per 10,000 inhabitants) from 1985 to 2019. Purple rectangles represent seasons chosen as test set, while the gray rectangle represents the season excluded from the analyses (season 2009–2010). All other seasons comprise the training set. **B**. Unnormalized weights used for the modified particle filter, with v1 shown in brown and v2 shown in green. For the weighting scheme v1, the weight assigned to particles was 1 if the projected incidence lies within the minimum and maximum observed incidence for the corresponding calendar week across the training seasons, and followed an exponential decrease when moving away from that minimum or maximum. For the weighting scheme v2, we used a normal distribution parameterized with the mean and the standard deviation of the observed incidence for the corresponding calendar week across the training seasons. **C**. Projection targets used for model performance evaluation: 1, 2, 3, and 4 week-ahead projections (w1, w2, w3, w4), peak intensity, and peak timing. Black (empty) dots denote data seen (unseen) at the time the projections are made, respectively.

Data about influenza activity in France from 1985 to 2019 (Fig 1A) were obtained from the French Sentinelles network (Réseau Sentinelles) [31], which is a volunteer-based information system of physicians created in France in 1984 and collecting real-time epidemiological data about different infectious diseases—among which influenza-like illness (ILI). In this surveillance system, ILI is defined as sudden fever above 39°C (102°F) with myalgia and respiratory signs. In this study, we model the national ILI incidence rate (rate per 10,000 inhabitants).

### Transmission model

We used a simple Susceptible-Infectious-Recovered (SIR) model to describe the transmission process in a closed population over an epidemic season under the assumption of homogeneous mixing. The system of differential equations for this model is:

$$\left\{\begin{array}{l} \frac{ds}{dt}=-\beta si\\ \frac{di}{dt}=\beta si-\gamma i\\ \frac{dr}{dt}=\gamma i \end{array}\right.$$

where *s*(*t*), *i*(*t*), and *r*(*t*) are the proportion of susceptible, infectious, and recovered individuals, *β* is the (constant) transmission rate, and 1/*γ* is the average infectious period (2.6 days [32]).

The basic reproduction number, i.e. the average number of secondary infections arising from an infected individual in a fully susceptible population, is *R*_0_ = *β*/*γ*.

The hidden state of the system included: 1) the number of susceptible individuals (S); 2) the number of infectious individuals (I); and 3) the basic reproduction number (*R*_0_).

At the beginning of each season, the latent variables were initialized as follows:

- The proportion of infectious individuals was drawn uniformly at random between 10–5 and 10–3, while the proportion of immune individuals was drawn uniformly at random between 0.0 and 0.3. The compartments S and I were then initialized accordingly.
- The reproduction number was drawn uniformly at random between 0.1 and 2.0.

### Observation model

Similarly to previous studies [10], we assumed a Gaussian observation process, whereby the observed incidence *x*_*obs*,*k*_ on week *k* conditional on the simulated incidence is given by:

$$x_{obs,k}\sim N\left(\mu =x_{sim,k},\sigma^{2}=\sigma_{obs,k}^{2}\right)$$

where *N*(*μ*, *σ*^2^) is a Gaussian distribution with mean *μ* and variance *σ*^2^, *x*_*sim*,*k*_ = *ρz*_*sim*,*k*_ is the simulated incidence on week *k*, with *z*_*sim*,*k*_ the simulated number of new infections on week *k* and *ρ* the reporting parameter (proportion of infected people that are captured by the surveillance system), and $\sigma_{obs,k}^{2}$ is computed as follows:

$$\sigma_{obs,k}^{2}=\xi_{1}+\xi_{2}x_{obs,k}^{2}$$

The parameters *ρ*, *ξ*_1_, and *ξ*_2_ were optimized independently for each Bayesian filter during the training phase (see *Model training and evaluation*).

### Bayesian filters

We evaluated the performance of four Bayesian filters: 1) the standard bootstrap particle filter (PF) [33,34]; 2) our modified bootstrap particle filter (mPF); 3) the ensemble Kalman filter (EnKF) [10]; and 4) the ensemble adjustment Kalman filter (EAKF) [10,35].

### Standard particle filter

Particle filters are used in the context of approximate inference for Hidden Markov Models (HMMs), which are models involving latent (hidden) variables that are observed through noisy measurements. Given their flexibility, they are used in a wide range of domains (see [33] for an in-depth introduction to the subject).

Particle filters rely on a cloud of weighted particles, where each particle represents a possible system state. The probabilistic model is defined via three components:

- An initial (prior) distribution, representing the hidden state of the system at time t = 0.
- A transition function, i.e. the function that describes the system dynamics (transmission model).
- An observation model, which describes how the noisy measurements relate to the hidden state.

In the bootstrap particle filter (PF) case, the particle’s weights are then used to resample the particles with replacement. The algorithm proceeds as follows [33,34]:

1. Initialization
  - Draw an initial set of particles from the initial distribution and assign equal weights to them: each particle represents a potential initial state of the system.
2. State update
  - Propagate each particle forward in time using the transition function.
  - Introduce some noise (regularization) to account for uncertainty and prevent degeneracy (the situation where only a few particles have significant weights).
3. Weight update
  - Use the observation model to evaluate the likelihood of the observed data given each particle’s state. This gives the weight to be assigned to each particle (*w*_*k*_).
4. Resampling
  - Resample particles with replacement according to their weights: particles with larger weights (i.e. representing trajectories that are closer to observations) are more likely to be selected multiple times, while those with smaller weights may end up being discarded altogether.
5. Repeat 2–4 for all time steps up to the current time.

After the filtering phase (i.e. after estimating the state of the system up to the current time), the cloud of particles can be used to make predictions beyond the current time point:

6. Forecasting
  - Simulate the future state of the system by propagating the particles forward in time using the transition function.
  - Repeat for the required number of time steps.
  - Compute summary statistics from the forecasted particles.

### Modified bootstrap particle filter (mPF)

When generating projections with a standard PF, the transmission model is used to update the particle trajectory but no weighting or resampling is done—since no observations are available for the future. The idea of the mPF is to continue weighting and resampling particles while generating projections: the weights assigned to the particles are computed using information extracted from training seasons.

The algorithm for the modified Particle Filter (mPF) is identical to the standard PF up until forecasting (step 6), which was replaced by the following step:

6. Forecasting (mPF)
  - Simulate the future state of the system by propagating the particles forward in time using the transition function.
  - Assign weights to each particle based on information extracted from the training seasons. In particular, the main idea is to give larger (lower) weights to trajectories that are closer to (far from) what was observed in the training seasons, respectively. See the description of the weighting schemes below.
  - Resample particles with replacement according to the new weights.
  - Repeat for the required number of time steps.
  - Compute summary statistics from the forecasted particles.

We tested two weighting schemes: v1 and v2 (see also Fig 1B).

The idea behind mPF_v1 was to give the maximum weight to all particles that fall between the minimum and maximum of incidence observed in training seasons, so that forecasted trajectories were compatible with trajectories observed in training seasons. The (unnormalized) weights for mPF_v1 are defined as follows:

$$w_{k}=\left\{\begin{array}{ll} \text{exp}\left(-\alpha \left(P_{k}-z_{k}\right)\right) & if\;z_{k}\leq P_{k}\\ 1 & otherwise\\ \text{exp}\left(-\alpha \left(z_{k}-Q_{k}\right)\right) & if\;z_{k}\geq Q_{k} \end{array}\right.$$

where *k* is the calendar week, *w*_*k*_ is the weight assigned to the particle, *z*_*k*_ is the projected incidence obtained from the particle, *P*_*k*_ and *Q*_*k*_ are respectively the minimum and maximum observed incidence for the training seasons, and *α* is a free parameter. On the other hand, mPF_v2 uses a Gaussian prior, giving more weight to particles that are closer to the mean of observed incidence. The weights for mPF_v2 are defined as:

$$w_{k}=N\left(z_{k}|\;\mu =m_{k},\sigma =\alpha s_{k}\right)$$

where *m*_*k*_ and *s*_*k*_ are the mean and the standard deviation of the observed incidence for calendar week *k*, and *α* is a free parameter.

For both versions of the mPF, the parameter *α* can be intuitively viewed as determining how ‘faithful’ to seasons in the training set the projections obtained from the mPF are allowed to be:

- For mPF_v1, a large *α* would result in a weight close to zero for any particle with predicted incidence not in the interval [*P*_*k*_, *Q*_*k*_]. Conversely, setting *α* = 0 would not constrain the projections at all.
- Similarly, for mPF_v2, setting *α* = 0 would assign a weight equal to zero to any particle with predicted incidence different from *m*_*k*_, while choosing a very large *α* would not constrain the projections.

### Ensemble filters

In contrast to the PF, the EnKF and EAKF assume a Gaussian prior and posterior distributions: the ensemble of particles are used to represent the mean and standard deviation of these distributions, and the two methods only differ with respect to how the posterior distribution is computed at each time step (see [10] and section *Implementation* for more details about the methods and their implementation).

### Implementation

When running the filters, we used 10,000 particles for the mPF and for the PF, and 3,000 particles for the two Kalman filters. According to previous analyses [10], filter performance does not improve significantly when using more than 3,000 particles.

Following [10,36], we used regularization (i.e. we added a small amount of noise to the basic reproduction number of each particle) when running the PF and the mPFs. The amount of noise (or regularization strength, *σ*) was optimized during the training phase (Table A in S1 Text and section *Model training and evaluation*). For the two Kalman filters, we used a multiplicative covariance inflation factor (*λ*) to counter filter divergence as described in [4,10,36]. The regularization step was followed by a check that all latent variables (S, I, and R0) were within valid bounds, with negative values clipped to 0 (S and I) or 0.1 (R0).

Finally, we followed [34] for the implementation of the PF and [10] for the implementation of the two Kalman filters. All analyses were performed in R [37].

### Baseline/Statistical models

We also compared the Bayesian filters to two baseline/statistical models. The first one (“baseline”) was a simple historical model, in which 2000 trajectories of ILI incidence were sampled from a truncated normal distribution, whose mean and variance were computed on the training seasons, for each week of the season. This model does not update based on recently observed data. The second model was a classical time-series model, the seasonal autoregressive integrated moving average model (“SARIMA”). The model was fitted to log-transformed data and forecasts were obtained by sampling 2000 trajectories of ILI incidence over the rest of the season, using the forecast package in R [38] and codes from [39].

### Model training and evaluation

We used data from 1985 to 2019 for a total of 34 influenza seasons: the pandemic season 2009–2010 was excluded from the analyses. We split this dataset into a training set consisting of 25 seasons—used to independently optimize each of the filters’ parameters—and a test set consisting of 8 seasons—used for performance evaluation on unseen data (Fig 1A). The 8 test seasons were considered as hypothetical “next” seasons and the predictions were made by only using data from the 25 training seasons (treated as observed seasons).

We considered each season as starting on week 30 (late July/beginning of August, depending on the season) of year *y* and ending on week 29 of year *y* + 1. Each filtering method was then run starting on week 40 of year *y* (beginning of October) and the process was repeated for a total of 33 weeks (until week 19 or 20 of year *y* + 1, corresponding to mid-May), each time using, as observations, the data up to—but not including—that week.

We used a broad grid search to evaluate, for each filter, different combinations of hyperparameters and selected the ones that maximized the performance—averaged over all targets and seasons—on the training set (see *Evaluation targets* and *Evaluation metrics* below, and Table A in S1 Text for the evaluated and optimized values).

For the modified particle filter, the summary statistics needed to define the weights used for the projections were computed using all the seasons in the training set—and none from the test set. As our setup provides richer information than available in real-time (the 8 test seasons are predicted using data from the 25 training seasons), the effect of reducing the number of seasons available to optimize the filters’ parameters and compute the summary statistics was explored in a sensitivity analysis. We randomly drew 5 subsamples of n seasons among the 25 training seasons, for n = 3, 4, … 9, 10, 15, 20 (50 subsamples in total). We optimized the filters’ parameters for each of the 50 subsamples, and predicted the 8 test seasons using these parameters and the summary statistics computed on the training seasons contained in each subsample.

### Evaluation targets

To evaluate the performance of the filtering methods we relied on 6 targets: the 1, 2, 3, and 4 week-ahead projections, the projected peak intensity (i.e. the projected maximum incidence for the season under consideration), and the projected peak timing (i.e. the projected week of maximum incidence) (Fig 1C).

### Evaluation metrics

Following recent literature on the evaluation of probabilistic projection accuracy [27,40,41], we primarily assessed the performance of the four filtering methods using the weighted interval score (WIS), which is defined as follows:

WISαP,y=1N+1212y−m+∑i=1Nαi2ui−li+li−yIy<li+y−uiIy>ui

Here *P* is the distribution representing the projections obtained from a model, *y* is the observation, *m* is the median projection, *α* is an *N*-dimensional vector of quantile levels, *l*_*i*_ and *u*_*i*_ are the *α*_*i*_/2 and 1 − *α*_*i*_/2 projection quantiles, and *I* is the indicator function.

The WIS metric penalizes not only prediction intervals that do not contain the observed data but also wide prediction intervals. Additionally, note that lower values correspond to better performance, with a *WIS* = 0 representing a perfect projection.

For our analyses we set *N* = 11, *α* = 0.02, 0.05, 0.1, 0.2, …, 0.9, and we used the average when combining scores from different weeks in a given season or when computing scores corresponding to multiple targets and/or seasons.

In addition, we also measured probabilistic forecast accuracy using the logarithmic score, defined as the predicted probability placed on the observed outcome [40]. Following [23], we computed modified logarithmic scores for the targets on the incidence scale such that predictions within +/- 100 cases per 100,000 inhabitants were considered accurate; i.e., given a model with a probability density function f(z) and true value z*, modified log $\text{score}=\text{log}\int_{z*-100}^{z*+100}f\left(z\right)dz$. For peak timing, predictions within +/- 1 week were considered accurate; i.e., modified log $\text{score}=\text{log}\int_{z*-1}^{z*+1}f\left(z\right)dz$. We truncated log scores to -10, to ensure all summary measures would be finite [23]; this rule was invoked for 2% of all scores. We report the exponentiated average log score.

We also computed the mean absolute error (MAE) to evaluate point forecasts (predictive medians). The MAE was defined as follows:

$$MAE=\frac{1}{n}\overset{n}{\underset{i=1}{\sum }}\left|y_{i}-z_{i}\right|$$

where *y*_*i*_ is the observed value at time *i*, *z*_*i*_ is the median projection obtained from a model for the same time point, and *n* is the number of time points considered.

## Results

**Fig 2:**
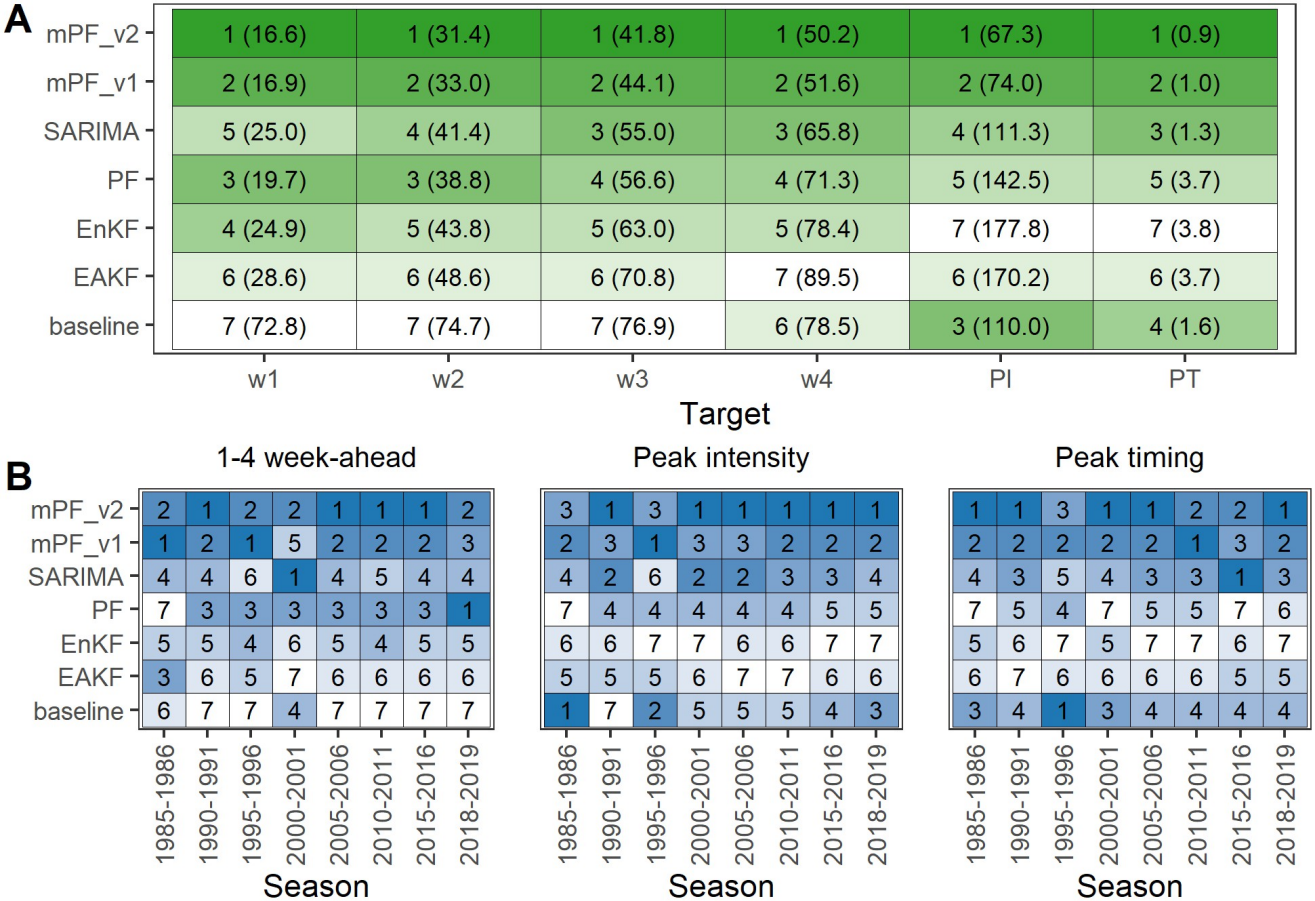
Model performance evaluation. **A**. Rank and WIS for the six projection targets averaged over all seasons in the test set: 1, 2, 3, and 4 week-ahead projections (w1, w2, w3, w4), peak intensity (PI), and peak timing (PT). **B**. Rank by target, for each season in the test set. In both panels each row represents a Bayesian filter, the text in each cell represents the rank (followed by the WIS in parentheses in panel A), and the color denotes model rank—with darker colors corresponding to lower WIS and therefore better performance. Baseline: simple historical model; EAKF: ensemble adjustment Kalman filter; EnKF: ensemble Kalman filter; mPF: our modified particle filter with weighting scheme v1 or v2; PF: standard particle filter; SARIMA: seasonal autoregressive integrated moving average model.

Fig 2A shows the WIS according to the projection target for the Bayesian filtering methods and the two baseline/statistical models tested in our analyses. We find that, when averaging over all test seasons, mPF_v2 performs better than any other method across all targets. The target for which the performance boost is most significant is the timing of the peak: mPF_v2 displays a WIS that is four times lower than that of the PF and the two Kalman filters. When considering the MAE or the log score as an evaluation metric instead of WIS, we also find that mPF_v2 provides better accuracy for all targets (Figs A and B in S1 Text). The parameter values of the different filters are shown in Table A in S1 Text. Values of *ξ*_1_ and *ξ*_2_ varied a lot across filters, leading to very different distributions of the observation process’ standard deviation (Fig C in S1 Text). Adding noise (regularization parameter σ > 0) in the standard and modified particle filters substantially improved their performance compared to filters without regularization (Fig D in S1 Text). In contrast, the parameter λ (covariance inflation factor) in Kalman filters had little influence on the overall performance (Fig D in S1 Text).

**Fig 3:**
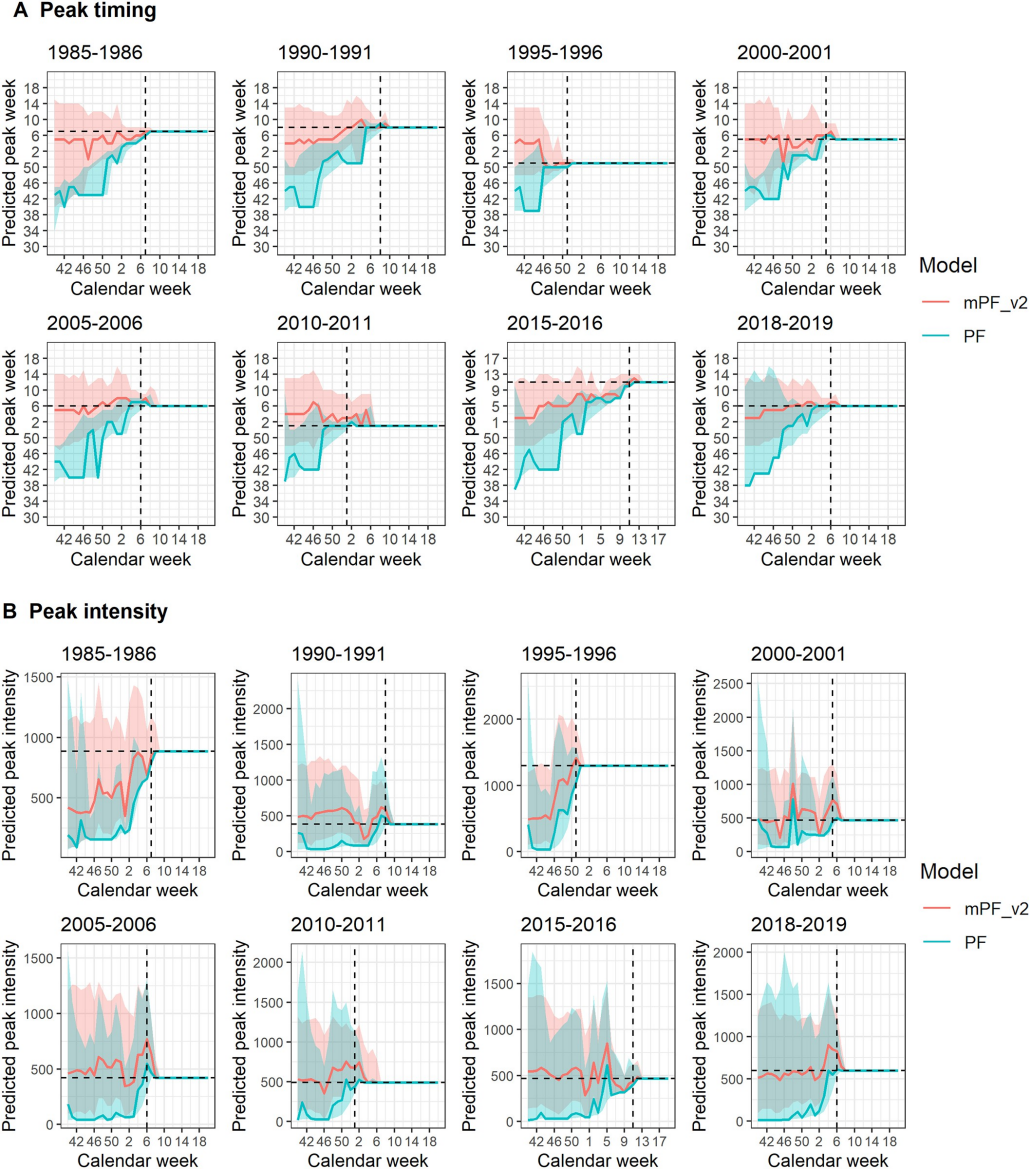
Predicted peak timing (A) and peak intensity (B) for the 8 test seasons, for the modified particle filter (mPF_v2) and the standard bootstrap particle filter (PF). Solid lines represent projection medians, light shaded areas represent 95% prediction intervals, and dashed lines represent the true values.

In Fig 2B, we report the WIS according to the projection target for each season in the test set: the performance boost of the mPF with respect to the PF is consistent across all test seasons, except for 1–4 week-ahead targets in 2018–2019 (Fig 2B and Fig E in S1 Text). The gain in performance is particularly high during the first weeks of a season, the differences between the mpF_v2 and the PF decreasing as we approach the peak (Fig 3 and Fig E in S1 Text). From the start of the season, the median predictions of the mPF for peak timing and intensity are much closer to the observed values—which almost always lie within the 95% prediction intervals -, whereas the PF systematically predicts an earlier peak, of lower intensity (Fig 3).

**Fig 4:**
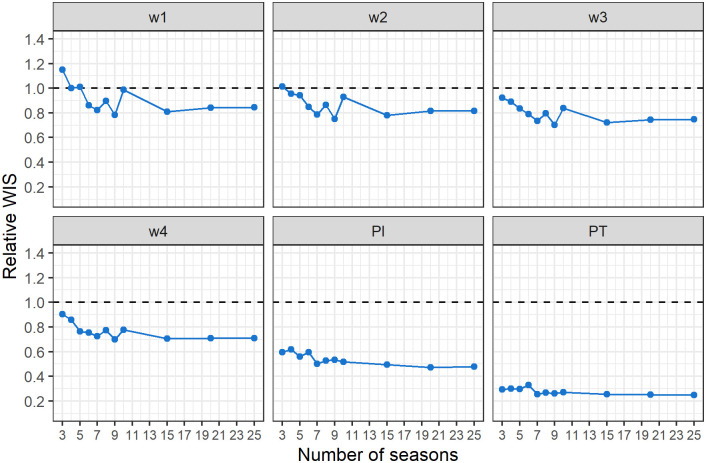
Comparison of the modified and standard particle filters. The six panels show the relative score of the mPF_v2 with respect to the standard PF—i.e. the WIS of the first divided by the WIS of the second—for each of the projection target and averaged over all seasons in the test set: 1, 2, 3, and 4 week-ahead projections (w1, w2, w3, w4), peak intensity (PI), and peak timing (PT). The x axis represents the number of seasons used when optimizing the filters’ parameters and computing the summary statistics for the mPF_v2. Values lower than 1 (dotted line) represent better performance of the mPF_v2.

In Fig 4, we show how the performance of the mPF method evolves as a function of the number of training seasons that are used when optimizing the filters’ parameters and computing the summary statistics needed to obtain its projections. In particular, we report the WIS of mPF_v2 relative to that of the PF for the 6 projection targets used in our analyses. We found that the mPF performance increases rapidly with the number of training seasons available to inform the projections: the mPF outperforms the PF when four training seasons are available for the 2 week-ahead target, and three training seasons for the 3-4 week-ahead targets and the seasonal targets (peak intensity and peak timing). Increasing the number of training seasons beyond ten slightly improves performance for week-ahead targets but the gain is marginal for seasonal targets.

## Discussion

The field of epidemic forecasting has experienced major developments in the last decade. Expectation is that epidemic forecast quality will increase if we develop a better understanding of the mechanisms driving the epidemic process and if we can learn from the mass of surveillance data that have been collected over the years. This has led to the development of two relatively distinct families of forecasting methods, based on mechanistic and statistical models, respectively. In general, the two approaches have been used separately, which we believe is suboptimal. For example, calibration of mechanistic models to data from the ongoing epidemic only misses opportunities to learn from trends observed in past epidemics. This may explain why, for influenza, statistical models have slightly outperformed mechanistic ones. Here, we proposed a simple approach that combines these two complementary paradigms by allowing the forecast of a mechanistic model to be weighted by patterns observed during epidemics from a training set.

We found that this approach substantially improved the forecast quality of mechanistic models for seasonal influenza. Compared to a standard particle filter, our filtering method predicts seasonal targets with a score (WIS) that is four times lower for peak timing and 2 times lower for peak intensity. The mPF shows that constraining projections according to the observations in the training seasons (i.e. accounting for the regularity that we expect to see in a non-pandemic influenza season) produces better results than simplifying the structure of the model (like Kalman filters do). We expect that the improvements will be more important when epidemic dynamics are characterized by regular epidemic patterns. Interestingly, in the specific context of seasonal influenza, we found that access to historical data for a few seasons only was sufficient to improve epidemic forecasts. Again, the number of seasons required to improve forecasts will likely depend on characteristics of seasonal epidemic being looked at: the more irregular it is, the larger the number of seasons required. While constraining projections according to the information extracted from the training seasons allows the mPF to outperform the other approaches studied in this paper, we note that the actual mechanisms presented here to achieve it (the v1 and v2 weighting schemes) do not follow from formal Bayesian derivations but remain heuristic in nature.

Although using historical data to inform influenza forecasts is more common for statistical/machine learning models than for mechanistic models [3,13,16–21,24,42,43] other studies also made use of past data to improve standard SIR models. For instance, the study by Ben-Nun et al [44] used data augmentation to make maximum use of prior data within a mechanistic framework. The data augmentation was a form of extrapolation in which future unobserved time points were assumed to take either a historical average or values equal to those in the most similar prior season. The study by Osthus et al [45] combined an SIR model for the disease-transmission process and a statistical model that accounted for systematic deviations (called discrepancy) between the mechanistic model and the data. It allowed forecasts to borrow discrepancy information from previously observed flu seasons, assuming that future influenza seasons will exhibit similar trajectories to past flu seasons. This model showed good performance in comparison with other forecasting models participating in the CDC’s 2015–2016 and 2016–2017 influenza forecasting challenge. These different approaches are promising ways of combining statistical and mechanistic models to improve forecasts performance.

Following recent studies on COVID-19, we primarily compared models based on WIS to assess probabilistic forecast accuracy [27,40,41]. The WIS is a proper score that approximates the continuous ranked probability score and can be interpreted as a generalization of the absolute error to probabilistic forecasts [40]. It is suitable for forecasts available in an interval or quantile format. MAE was also computed to assess point forecast error. Both scores agreed that the modified particle filters were performing best. In addition, we also measured probabilistic forecast accuracy using the logarithmic score, although it was reported to be less robust than the CRPS [46]. This proper score can be applied when the full predictive distribution is available. Similarly to what was previously done for the CDC *Flusight* challenge [23], we used a modified log score which counts probability mass within a certain tolerance range. It offers a more accessible interpretation but has the disadvantage of being improper [40,47,48] which prompted the CDC to move to the proper single-bin log score in 2019/2020 (applied to forecasts consisting of binned predictive distributions, which was not the case in our study)—before moving to WIS in subsequent years to evaluate forecasts in quantile format [43]. Interestingly, the log score yielded results comparable to the WIS, which is in line with a recent study comparing various scores and showing that despite some differences, the WIS and the log score agree on which are the best models [40].

In some situations, we expect that our approach might worsen forecasts. This is the case when the current epidemic is expected to be radically different from past epidemics, for example during influenza pandemics. In such a scenario, we recommend using a mechanistic model without relying on trends observed during past seasonal epidemics. One could track the degree of similarity between the current epidemic and past epidemics, to decide on which type of model to use [45].

We performed our evaluation using a simple mechanistic model with an SIR structure. In future developments, we aim to evaluate if forecast quality improves when including more covariates in the model such as climatic information [49], circulating virus [50] and mobility/school holidays data [51,52]. In addition, in our analysis, the reporting parameter *ρ* was constant across all test seasons, which might limit performance if the true reporting rate varies over time. An extension of this work could include *ρ* as a parameter that varies by season, although it might be difficult to estimate the reporting parameter at the start of an influenza season. Of note, our analysis was performed retrospectively, using consolidated data, and therefore did not produce pseudo-prospective forecasts based on data reported in real-time. Since delays in data availability or data revisions after their publication can increase the forecast error, our study may tend to overestimate the performance of the forecasting models, compared to what would be observed in real time. However, this overestimation would likely affect all models indistinctly and our conclusions would likely be unchanged. We also acknowledge that due to the single train-test split scheme that we used, the number of test seasons (N = 8) was limited. However, the results were consistent across the 8 test seasons, underscoring the robustness of the study findings.

Finally, we used French ILI syndromic surveillance data to model influenza epidemic dynamics. Such syndromic surveillance is an imperfect proxy of influenza activity as only a proportion of influenza infections are detected and other respiratory viruses may cause ILI. However, past analysis has shown that French ILI syndromic surveillance exhibited similar epidemic dynamics as that reported by French virologic surveillance, with a coefficient of correlation between the two time series of 0.85 and an average time lag between epidemic peaks of 0.22 weeks [51]. Our study focused on the pre-COVID-19 period, but we acknowledge that the use of syndromic surveillance to study influenza is more challenging in a context where COVID-19 is now also circulating. The expected co-circulation of SARS-CoV-2 and influenza during the winter season in the Northern Hemisphere (when climatic conditions are favorable for the transmission of both pathogens [53,54]) will bring new challenges for forecasting, including questions regarding ILI cases identification (due to symptomatic similarities between the two diseases), changing healthcare seeking behaviors, and possible interactions between the two viruses. Relying on different data sources that are more specific to influenza (such as laboratory-confirmed influenza hospital admissions as in [43]) or developing modelling approaches that account for co-circulating pathogens might be interesting avenues for improvement.

In conclusion, we developed a new approach for epidemic forecasting that integrates the key strengths of the statistical approach into the mechanistic modelling framework. This method is currently being tested in real-time to provide short-term forecasts in France.

## Supporting information

S1 Text Supplementary material. (PDF)
