# Supplementary material for: Integrating information from historical data into mechanistic models for influenza forecasting
Source: PLoS Comput Biol. 2024 Oct 30;20(10):e1012523. doi: 10.1371/journal.pcbi.1012523 (PMC11524484; doi:10.1371/journal.pcbi.1012523)
Supplement: S1 Text — (PDF) [file pcbi.1012523.s001.pdf]

Integrating information from historical data into mechanistic models for influenza forecasting

Alessio Andronico[1#], Juliette Paireau[1,2#*], Simon Cauchemez[1]

1 Mathematical Modelling of Infectious Diseases Unit, Institut Pasteur, Université Paris Cité, UMR2000 CNRS
2 Infectious Diseases Department, Santé publique France, Saint-Maurice, France

#: Equal contribution

*juliette.paireau@pasteur.fr

S1 Text. Supplementary material

This document contains Supplementary Figures A to E and Supplementary Table A.

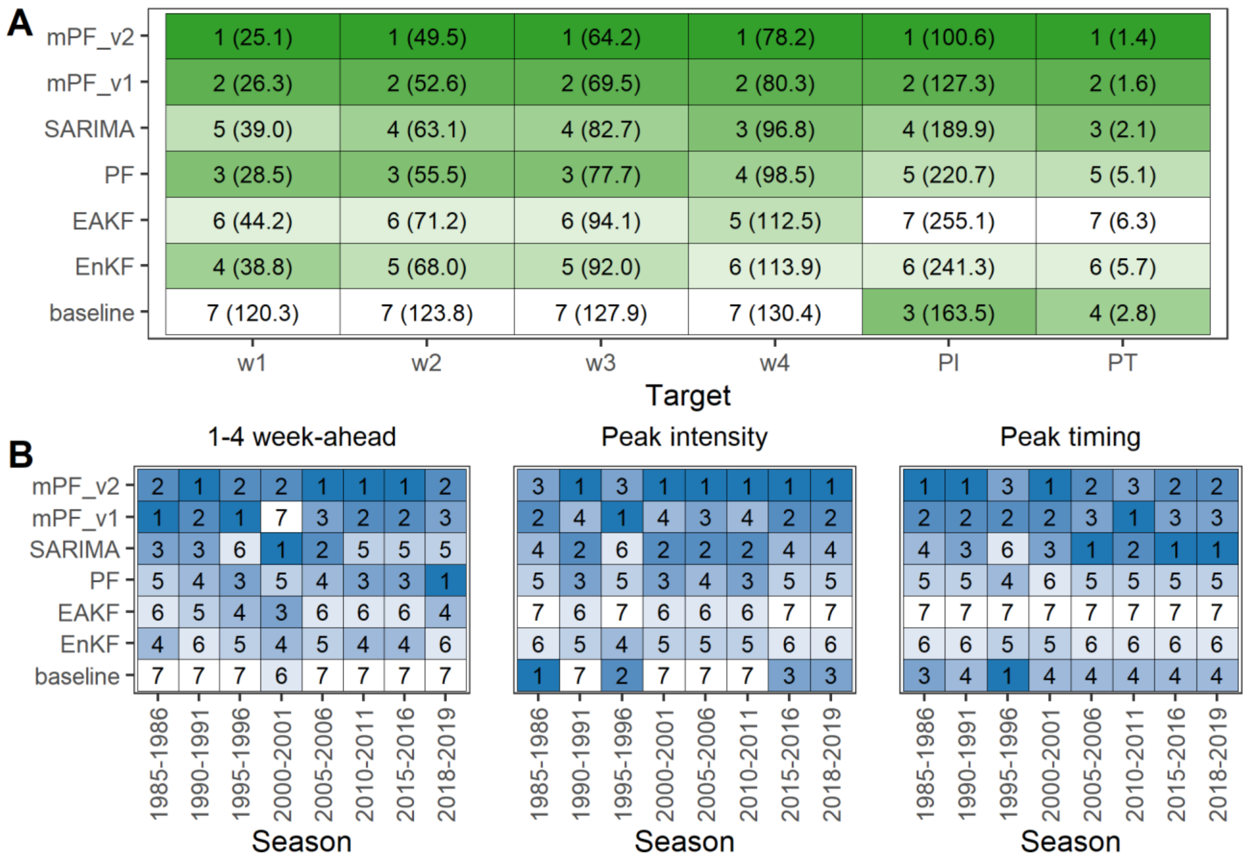

Fig A. Model performance according to the mean absolute error (MAE) metric. A. Rank and MAE for the six projection targets averaged over all seasons in the test set: 1, 2, 3, and 4 week-ahead projections (w1, w2, w3, w4), peak intensity (PI), and peak timing (PT). **B.** Rank and MAE averaged over all prediction horizons, by target, for each season in the test set. In both panels each row represents a Bayesian filter, the text in each cell represents the rank (followed by the MAE in parentheses in panel A), and the color denotes model rank - with darker colors corresponding to lower MAE and therefore better performance. Baseline: simple historical model ; EAKF: ensemble adjustment Kalman filter ; EnKF: ensemble Kalman filter ; mPF: our modified particle filter with weighting scheme v1 or v2 ; PF: standard particle filter ; SARIMA: seasonal autoregressive integrated moving average model.

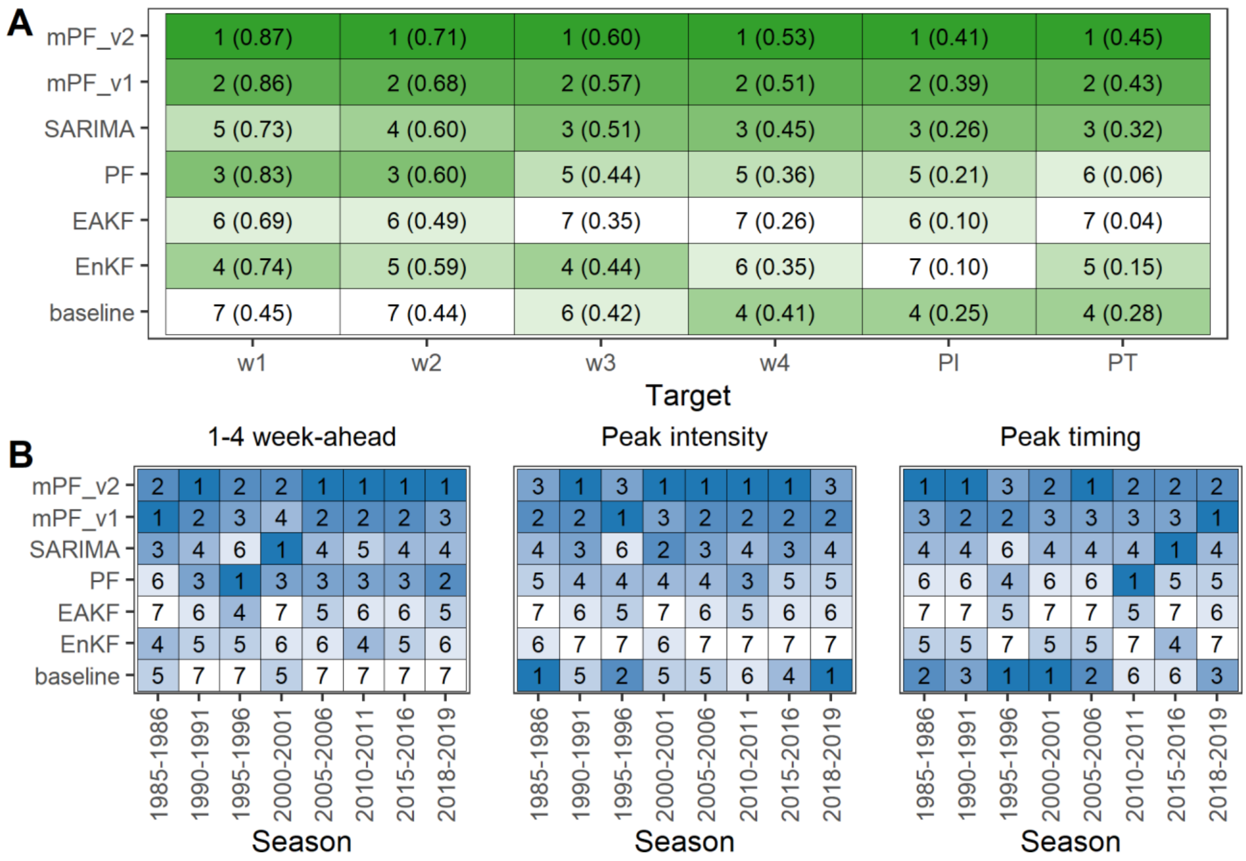

Fig B. Model performance according to the exponentiated average log score ("average score"). A. Rank and average score for the six projection targets over all seasons in the test set: 1, 2, 3, and 4 week-ahead projections (w1, w2, w3, w4), peak intensity (PI), and peak timing (PT). **B.** Rank and average score over all prediction horizons, by target, for each season in the test set. In both panels each row represents a Bayesian filter, the text in each cell represents the rank (followed by the average score in parentheses in panel A), and the color denotes model rank - with darker colors corresponding to higher average score and therefore better performance. Baseline: simple historical model ; EAKF: ensemble adjustment Kalman filter ; EnKF: ensemble Kalman filter ; mPF: our modified particle filter with weighting scheme v1 or v2 ; PF: standard particle filter ; SARIMA: seasonal autoregressive integrated moving average model.

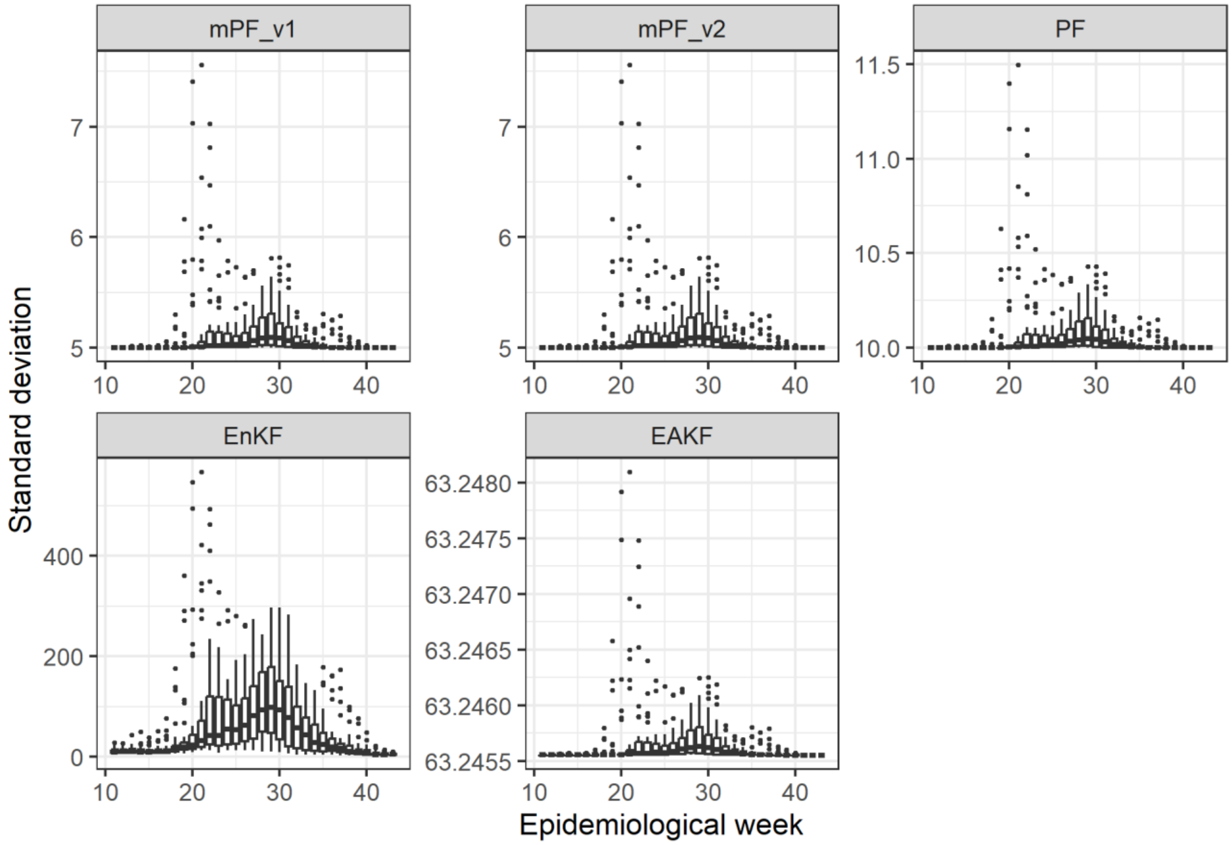

Fig C. Distributions of $\sigma_{obs,k}$ (standard deviation of the observation process) for each Bayesian filter, by epidemiological week, over all seasons in the dataset. EAKF: ensemble adjustment Kalman filter ; EnKF: ensemble Kalman filter ; mPF: our modified particle filter with weighting scheme v1 or v2 ; PF: standard particle filter.

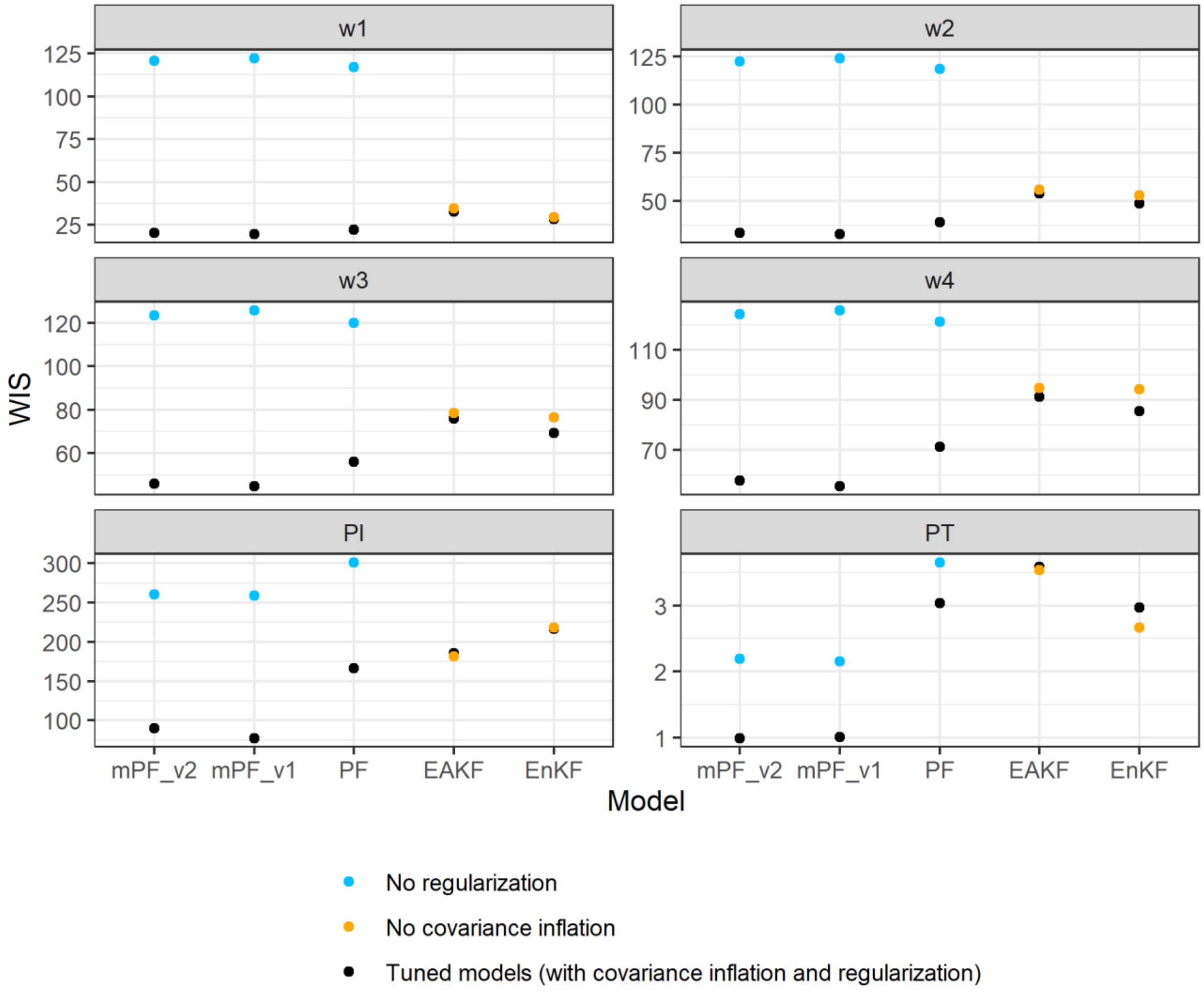

Fig D. Comparison of the performance of particle filters with (σ > 0) or without (σ = 0) regularization, and Kalman filters with (λ > 1) or without (λ = 1) covariance inflation, based on the WIS (weighted interval score), for the six projection targets: 1, 2, 3, and 4 week-ahead projections (w1, w2, w3, w4), peak intensity (PI), and peak timing (PT). EAKF: ensemble adjustment Kalman filter ; EnKF: ensemble Kalman filter ; mPF: our modified particle filter with weighting scheme v1 or v2 ; PF: standard particle filter.

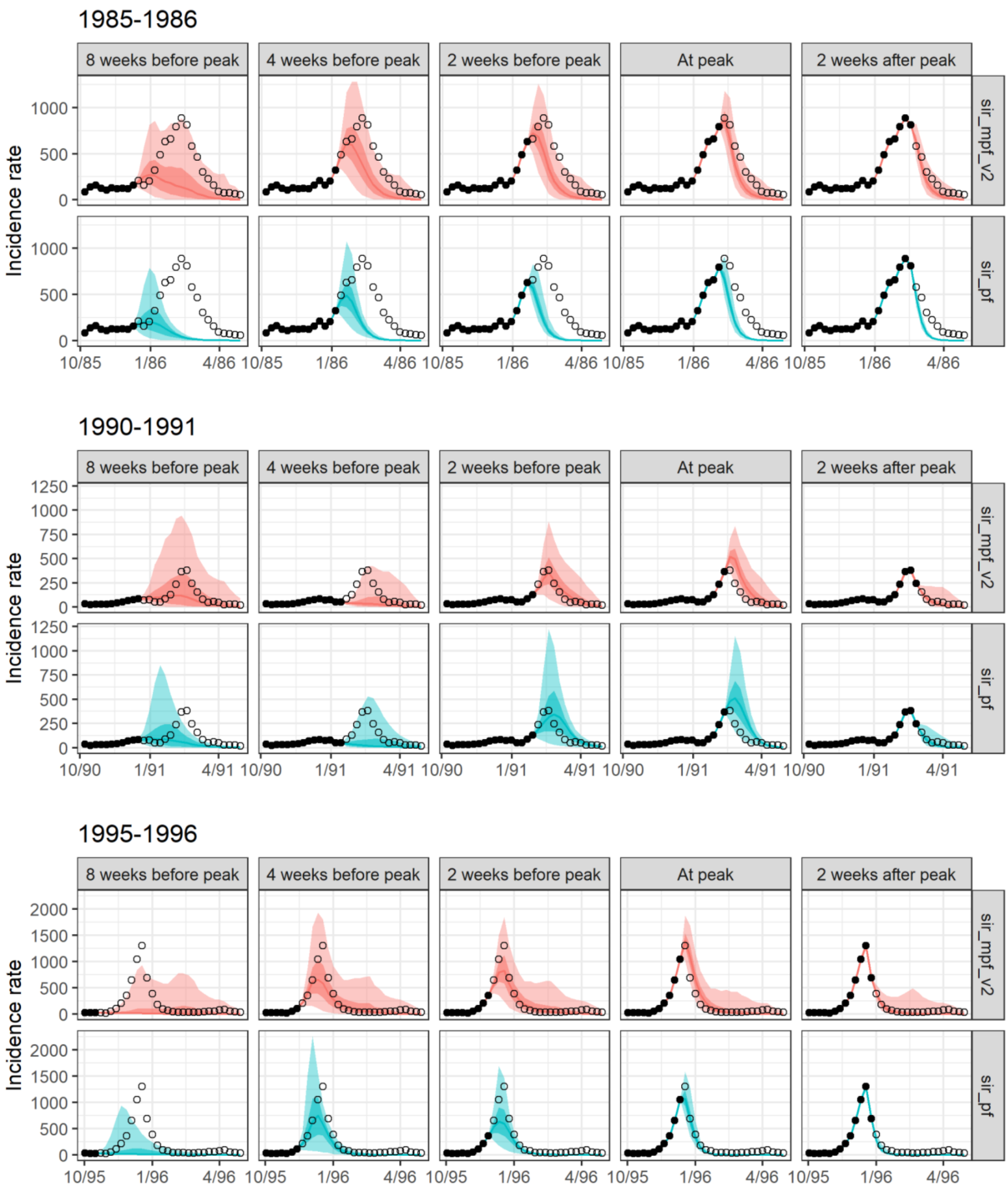

1985-1986
8 weeks before peak
4 weeks before peak
2 weeks before peak
At peak
2 weeks after peak
sir_mpf_v2
sir_pf
Incidence rate
0
500
1000
10/85
1/86
4/86
1990-1991
8 weeks before peak
4 weeks before peak
2 weeks before peak
At peak
2 weeks after peak
sir_mpf_v2
sir_pf
Incidence rate
0
250
500
750
1000
1250
10/90
1/91
4/91
1995-1996
8 weeks before peak
4 weeks before peak
2 weeks before peak
At peak
2 weeks after peak
sir_mpf_v2
sir_pf
Incidence rate
0
500
1000
1500
2000
10/95
1/96
4/96

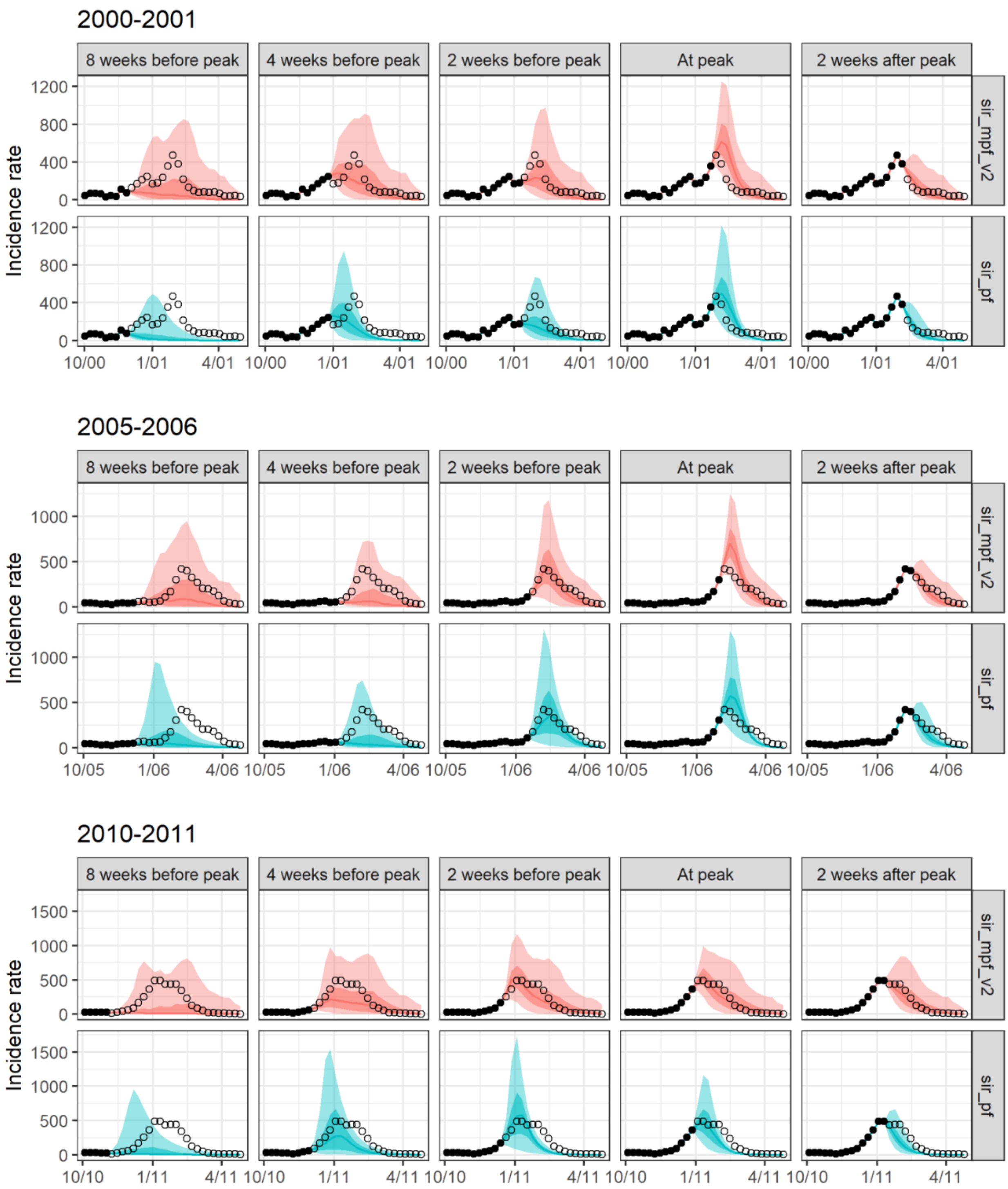
2000-2001
8 weeks before peak
4 weeks before peak
2 weeks before peak
At peak
2 weeks after peak
Incidence rate
1200
800
400
0
sir_mpf_v2
sir_pf
10/00
1/01
4/01
2005-2006
1000
500
10/05
1/06
4/06
2010-2011
1500
10/10
1/11
4/11

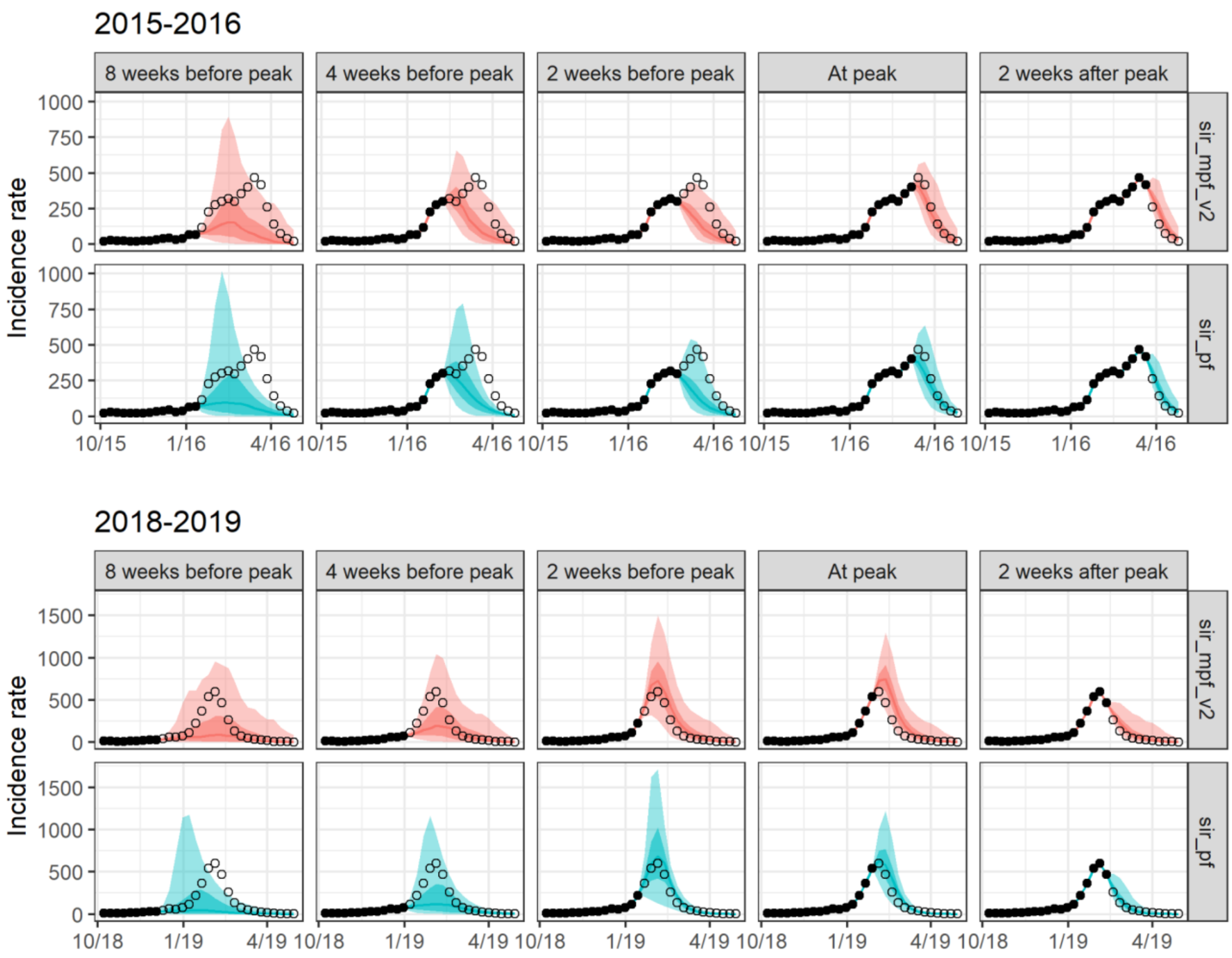

Fig E. Retrospective projections for all the seasons in the test set. The top line of each season corresponds to projections obtained with the modified particle filter (mPF_v2), while the bottom line of each season corresponds to projections obtained using the standard bootstrap particle filter (PF). From left to right, the five columns show projections made 8 weeks before the epidemic peak (week of maximum incidence), 4 weeks before the epidemic peak, 2 weeks before the epidemic peak, on the week of the epidemic peak, and 2 weeks after the epidemic peak. Black dots denote data assimilated by the filtering methods and informing their projections, while empty dots represent data unseen at the time the projections were made. Lines represent projection medians, light shaded areas represent 95% credible intervals, while darker areas represent 50% credible intervals.

Model	ξ_1	ξ_2	ρ	σ	α	λ
**mPF_v1**	10, **25**, 100, 200, 500, 1000, 2000, 4000	10^{-7}, 10^{-6}, $\mathbf{10^{-5}}$, 10^{-4}, 10^{-3}, 10^{-2}, 10^{-1}	0.1, **0.3**, 0.5	0.05, **0.1**, 0.2, 0.4	0.001, **0.01**, 0.01, 1	NA
**mPF_v2**	10, **25**, 100, 200, 500, 1000, 2000, 4000	10^{-7}, 10^{-6}, $\mathbf{10^{-5}}$, 10^{-4}, 10^{-3}, 10^{-2}, 10^{-1}	0.1, **0.3**, 0.5	0.05, **0.1**, 0.2, 0.4	0.5, 1, **2**, 3	NA
**PF**	10, 25, **100**, 200, 500, 1000, 2000, 4000	10^{-7}, 10^{-6}, $\mathbf{10^{-5}}$, 10^{-4}, 10^{-3}, 10^{-2}, 10^{-1}	**0.1**, 0.3, 0.5	0.05, 0.1, **0.2**, 0.4	NA	NA
**EnKF**	**10**, 25, 100, 200, 500, 1000, 2000, 4000	10^{-7}, 10^{-6}, 10^{-5}, 10^{-4}, 10^{-3}, 10^{-2}, $\mathbf{10^{-1}}$	0.1, **0.3**, 0.5	NA	NA	1.03, 1.05, **1.07**
**EAKF**	10, 25, 100, 200, 500, 1000, 2000, **4000**	$\mathbf{10^{-7}}$, 10^{-6}, 10^{-5}, 10^{-4}, 10^{-3}, 10^{-2}, 10^{-1}	**0.1**, 0.3, 0.5	NA	NA	**1.03**, 1.05, 1.07

Table A. Parameters for the Bayesian filters used in the analyses. The table shows the values that were tested using a broad grid search. The values shown in bold are those that maximized performance on the training set and were selected to evaluate the models on the test set. ξ_1 and ξ_2 are used to define the variance of the observation process, ρ is the reporting parameter, σ is the regularization strength used for the PF and the mPF, α is the parameter defining the weights used by the mPF while generating projections, and λ is the multiplicative inflation factor used for the Kalman filters. NA (Not Applicable) entries represent parameters that are not relevant for that filter. EAKF: ensemble adjustment Kalman filter ; EnKF: ensemble Kalman filter ; mPF: our modified particle filter with weighting scheme v1 or v2 ; PF: standard particle filter.
